# Effect of Nudge Interventions in Real-World Kiosks on Consumer Beverage Choices to Promote Non-Sugar-Sweetened Beverage Consumption

**DOI:** 10.3390/nu17152524

**Published:** 2025-07-31

**Authors:** Suah Moon, Seo-jin Chung, Jieun Oh

**Affiliations:** 1 Department of Nutritional Science and Food Management, Ewha Womans University, Seoul 03760, Republic of Korea; msa2926@ewhain.net (S.M.); sc79d@ewha.ac.kr (S.-j.C.); 2 College of Science & Industry Convergence, Ewha Womans University, Seoul 03760, Republic of Korea

**Keywords:** sugar-sweetened beverages, kiosks, nudge, carbonated beverages, healthy eating, consumer behaviors

## Abstract

**Background/Objectives**: Excessive sugar intake through sugar-sweetened beverages (SSBs) has raised global concerns due to its association with various health risks. This study evaluates the effectiveness of nudges—in the form of order placement, variety expansion, and a combination of both—in promoting non-SSB purchases at self-service kiosks, a key environment for SSB consumption. **Methods**: This study was conducted using a real-world kiosk at food and beverage outlets in South Korea from 28 May to 12 July, 2024. A total of 183 consumers aged 19 to 29 participated in this study. A single kiosk device was used with four screen layouts, each reflecting a different nudge strategy. Participants were unaware of these manipulations when making their purchases. After their purchases, participants completed a survey. All data were analyzed using IBM SPSS Statistics for Windows, Version 29.0. **Results**: Females reported significantly higher positive attitudes, preferences, and perceived necessity regarding nudges compared to males. In particular, both the single (variety) and combination (order and variety) nudges received positive responses from females (*p* < 0.001). The combination nudge significantly increased non-SSB purchases compared to the control (*p* < 0.05) and single (order) nudge groups (*p* < 0.01), which suggests that combination nudge is effective in promoting healthier beverage choices. Females were also more likely to purchase non-SSBs than males (*p* < 0.05). **Conclusions**: The findings suggest that the combination nudge strategy effectively promotes healthier beverage choices in real kiosk settings. Notably, females demonstrate significantly higher positive attitudes, preferences, and perceived necessity regarding nudges compared to males, and are also more likely to purchase non-SSBs. These findings offer valuable insights for real-world applications aimed at encouraging healthier consumption behaviors.

## 1. Introduction

The global trend of excessive sugar consumption through sugar-sweetened beverages (SSBs) has raised public health concerns given its contribution to various health issues [1,2]. In the United States, the per capita consumption of carbonated beverages has increased nearly fivefold over the past 50 years [3]. Similarly, in Brazil, the consumption of carbonated beverages rose approximately 400-fold between 1974 and 2003 [4]. Each additional serving of SSBs is associated with a 27%, 19%, and 10% increased risk of type 2 diabetes, CVD, and all-cause mortality, respectively [5]. Sugar intake is also a primary cause of dental caries due to its role in acid production [6].

Notably, beverages are the leading source of sugar intake from processed foods among Koreans, with individuals aged 19–29 years consuming the highest proportion (35.5%) of their sugar from beverages [7]. Among this age group, carbonated beverages account for the majority of sugar intake. According to the eighth Korea National Health and Nutrition Examination Survey, males and females aged 19–29 years consumed 147.7 ± 19.5 g and 102.4 ± 11.3 g of carbonated beverages per day, respectively—the highest levels among all age groups [8]. Additionally, Park found that carbonated beverages were the most frequently consumed sugary food among 296 Korean university students, with males and females consuming them approximately twice and three times a week, respectively [9]. Among the Korean population, Kwak et al. identified a significant association between excessive SSB consumption and hypertension [10]. Given this finding and the various negative health impacts described above, there is an urgent need for actionable strategies to reduce SSB consumption among young consumers in beverage-focused environments.

Self-service technologies, especially kiosk systems, have been rapidly adopted in fast-food chains like McDonald’s [11]. These systems enhance user convenience by reducing order and wait times [12]. However, it is important to note that fast-food restaurants are notable sites of SSB consumption, where beverages like carbonated drinks are often bundled with meals and offered with free refills, further encouraging SSB consumption [13,14]. In South Korea, individuals in their twenties predominantly purchase these drinks at fast-food restaurants [15]. Therefore, fast-food kiosks can be considered a key point of sale for beverage selection in the modern food service environment.

Nudge theory, introduced by Thaler and Sunstein, refers to any aspect of choice architecture that influences behavior without restricting options or changing incentives [16]. Nudges have been widely applied in various social behavior studies to promote sustainable actions [17,18,19] and have proven particularly effective in encouraging healthier choices in the food domain [20,21,22,23]. The World Health Organization recommends several nudge strategies to promote healthier eating habits, such as positioning healthier options at eye level (placement), increasing their variety (availability), and highlighting them for greater visibility (contrast). Other strategies include enhancing the appeal of healthier choices (presentation), using attractive names or symbols (descriptives and semiotics), providing visual cues (prompts), and setting healthier options as the default choice (default) [24].

Attitudes toward nudging can be explained through dual process theory [16]. This theory posits that human cognition operates through two distinct systems: an intuitive and automatic system, and a reflective and deliberative one [25]. Kahneman termed these systems System 1 and System 2, respectively [26]. System 1 processes information rapidly and intuitively, without requiring conscious comparison. For example, strategies such as placing healthier food options in more prominent positions primarily engage System 1 [27]. In contrast, System 2 involves deliberate comparison and cognitive effort, and is activated by providing nutritional information that supports conscious decision-making [28]. Accordingly, this study draws on both the WHO’s practical guidelines and the theoretical framework of dual process theory to examine the effectiveness of two nudging strategies: (1) a placement nudge that encourages intuitive decision-making by adjusting the position of healthier beverages, and (2) a variety nudge that promotes reflective comparison by increasing the availability of non-SSB options with explicit nutritional cues—such as ‘100% freshly squeezed’ and ‘zero’ labels. These strategies—particularly placement and variety nudges—have been shown to effectively influence healthier consumer choices in a range of consumption environments.

Anesbury et al. noted that when purchasing food online, the absence of physical store navigation significantly shortens decision-making times [29]. Therefore, placing items at the top of the page is an effective way to influence consumer choices. Schmidtke et al. demonstrated that placing zero-calorie cola as the first option on fast-food kiosks significantly increased its sales [30]. Similarly, Dayan and Bar-Hillel observed that menu items placed at the edges received more attention and were selected at a higher rate compared to those in the middle, which were more difficult to access [31]. Based on this evidence, this study incorporates an order nudge by positioning non-SSBs as the first option on the kiosk interface. Ensaff highlighted that increasing product variety enhances visibility, making products more noticeable and easier to choose, aligns with social norms, and boosts consumer preferences for widely recognized options [32]. Similarly, Hollands et al. suggested that expanding the availability of products increases exposure, fostering greater consumer acceptance and preference [33]. This approach can be applied to promote healthier food choices. Therefore, this study also employs a variety nudge by increasing the number of non-SSB options available on kiosks.

Yet, as human cognitive capacity to receive and process information is limited, excessive stimuli can influence decision-making outcomes [34]. In line with this view, Howley, P., & Ocean, N. suggested that combining two nudges may weaken their individual effects due to psychological crowding out, but if such interference does not occur, the combined nudges could be used more positively and effectively; therefore, given the lack of empirical research grounded in this theoretical perspective, further investigation into the effects of combined nudges is warranted [35]. Relevant empirical studies include Hua et al., who found that combining two or more nudges results in a significantly greater increase in healthy food choices compared to applying a single nudge [36], and a study by Yi et al. of university students demonstrated that combined nudges significantly increase long-term healthy food choices [27]. Therefore, this study also adopted a combination kiosk, which integrated both an order nudge and a variety nudge. Building on these findings, this study aimed to examine whether a combination of order and variety nudges could produce synergistic effects in a real-world digital food purchasing environment, such as fast-food kiosks. By applying this dual strategy in a context where consumer decisions are increasingly mediated by self-service technology, we sought to assess its practical utility for guiding healthier beverage choices.

To achieve public health objectives in modern society, it is crucial to address the issue of excessive sugar intake, particularly by implementing changes in the consumption of sugar-sweetened beverages (SSBs), which serve as a primary source of added sugars. This study aims to identify effective nudging strategies that encourage the transition from SSBs to healthier beverage choices. To assess the impact on actual consumer behavior, this study introduced modifications to the product sales environment using real-world kiosks.

## 2. Materials and Methods

### 2.1. Experiment Design

This study was conducted using a single kiosk at food and beverage outlets located in Seoul, South Korea. To identify commonly sold beverages and their kiosk arrangements in Korean fast-food restaurants, we visited eight franchise outlets, all of which used kiosk systems and offered similar hamburger-set menus. Based on these observations, 14 beverage types were selected for this study (as enumerated in the section below), with the average price of each beverage brand used as the price point. The beverage images used in the kiosk were generated with DALL·E 3 software (OpenAI, San Francisco, CA, USA) and designed to mirror the layout and composition of images typically found in fast-food restaurant kiosks.

### 2.2. Kiosk Screen Configurations

**Figure 1:**
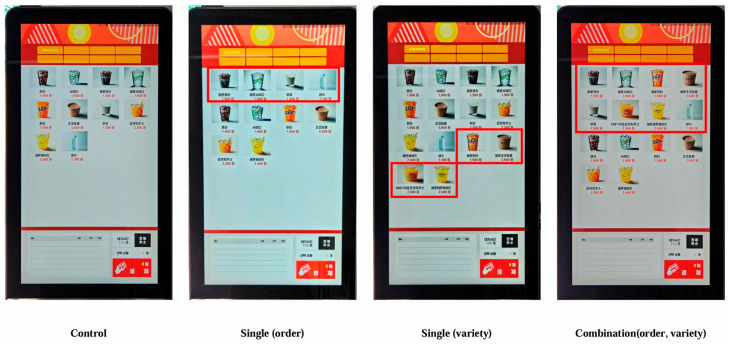
Beverage kiosks used in the experiment. Red boxes, shown only in the intervention conditions, highlight the non-SSBs included in each kiosk layout. The images are photographs of the actual kiosks used during the experiment and contain beverage names written in Korean. The English equivalents are as follows—Cola: 콜라, lemon–lime soda: 사이다, orange soda: 환타, chocolate drink: 초코음료, orange juice: 오렌지주스, lemonade: 레몬에이드, Diet Cola: 제로콜라, Diet lemon–lime soda: 제로사이다, milk: 우유, bottled water: 생수, Diet orange soda: 제로환타, Diet chocolate drink: 제로초코음료, 100% freshly squeezed orange juice: 100% 착즙 오렌지주스, Diet lemonade: 제로레몬에이드.

**Figure 2:**
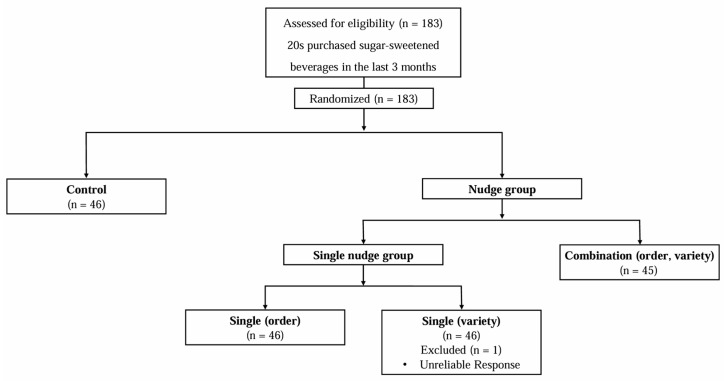
Research design.

The kiosk screen settings were altered to create four distinct groups, and the screen layouts for each group are shown in Figure 1. The control kiosk featured 10 beverage types commonly sold across the eight fast-food outlets (6 SSBs: Cola, lemon–lime soda, orange soda, chocolate drink, orange juice, lemonade; and 4 non-SSBs: Diet Cola, Diet lemon–lime soda, milk, bottled water), listed in the order of their actual arrangement in the kiosk. The order kiosk offered the same beverages as the control kiosk but applied a placement nudge, positioning the non-SSBs at the front. The variety kiosk applied a variety expansion nudge by adding four non-SSBs (Diet orange soda, Diet chocolate drink, 100% freshly squeezed orange juice, and Diet lemonade) at the end of the selection in the control kiosk configuration. The combination kiosk combined both nudges, placing all non-SSBs at the front. Participants were assigned numeric IDs and randomly allocated into one of four kiosk conditions using a pre-generated assignment list created via a random assignment program (https://www.randomizer.org (accessed on 22 May 2024)). Each incoming participant was exposed to the kiosk configuration corresponding to their assigned ID. The number of participants and the sex distribution were similar across the four groups, with approximately 19% male and 81% female in each. The participant flow is presented in Figure 2.

According to the Labeling Standards for Foods, etc. (2024) issued by the Ministry of Food and Drug Safety (MFDS) of Korea [37], beverages containing less than 0.5 g of sugar per 100 mL are permitted to be labeled as sugar-free. Based on this criterion, diet beverages—including Diet Cola, Diet lemon–lime soda, Diet orange soda, Diet chocolate drink, and Diet lemonade—as well as bottled water, were classified as non-sugar-sweetened beverages (non-SSBs) in this study. In addition, products may be labeled as no added sugar or unsweetened if they (1) do not contain added sugars, (2) do not contain sugar substitutes used as functional replacements, (3) do not include ingredients with added sugars, (4) do not contain ingredients whose sugar content has increased due to concentration or drying, and (5) are not processed in ways—such as enzymatic hydrolysis—that increase the final sugar content of the food. Accordingly, 100% freshly squeezed orange juice and milk were also categorized as non-SSBs.

### 2.3. Experimental Procedure

Prior to participation, participants were given a brief explanation of this study at the food and beverage outlet. To minimize potential bias in beverage selection, partial deception was employed, a fictitious study title (Study on Beverage Kiosk Usage and Beverage Consumption Patterns Among Consumers in Their 20s) was presented instead of the actual research title. Participants were informed that the purpose of this study was to examine general beverage purchasing behaviors at kiosks, without disclosing the specific objective of evaluating the effects of nudging interventions on non-SSB choices. Participants were blind to their assigned kiosk condition and received no information about the intervention or group allocation prior to purchase.

Participants were then instructed to imagine being at a fast-food outlet and to purchase a beverage they would like to drink using their credit card at the kiosk. If the desired product was unavailable, they were advised to choose a similar product or one of the same type. Immediately after completing their purchase, a full debriefing was conducted. Participants were informed of the actual study title and objectives, as well as the specific kiosk configuration they had been assigned to and the details of all nudge interventions used in this study. At that time, they were clearly advised that their continued participation was entirely voluntary and that they could withdraw from this study or request the deletion of their data if they no longer wished to participate. Only those who provided informed consent after the debriefing proceeded to complete the subsequent online survey.

### 2.4. Participants

This study involved 183 consumers aged between 19 and 29 years. To recruit participants, recruitment notices were posted on buildings at Ewha Womans University and on online university student communities. Eligible participants were those who had consumed sugar-sweetened beverages (SSBs) within the past three months and provided written informed consent. A minimum sample size of 180 was determined using G*Power (version 3.1.9.7), based on a statistical power of 0.95, an alpha (α) level of 0.05, four comparison groups, and an anticipated dropout rate of 10%. Recruitment and data collection were conducted from 28 May to 12 July 2024. After excluding one incomplete response (0.5%), a total of 182 valid responses (99.5%) were retained for analysis. This study was approved by the Institutional Review Board (IRB) of Ewha Womans University (IRB No.: ewha-202404-0029-02).

### 2.5. Survey Instrument

#### 2.5.1. Demographic Profile and Baseline State

Participants’ demographic characteristics were collected, including sex, age, marital status, household type, monthly income or allowance (in KRW 10,000), height, weight, and current weight management status [38,39]. To assess baseline states, participants self-reported levels of thirst, hunger, and fatigue on a 7-point Likert scale (1 = strongly disagree, 7 = strongly agree). The time since participants had last eaten or drunk was also recorded on a continuous scale [40,41,42].

#### 2.5.2. Beverage Kiosk Purchasing Behavior

To assess participants’ typical usage behaviors with beverage kiosks, satisfaction with prior experiences, the extent of pre-purchase decision-making, and experiences of impulsive purchases were measured using a 7-point Likert scale (1 = strongly disagree, 7 = strongly agree) [43,44].

#### 2.5.3. Effect of Nudge Interventions in Beverage Kiosks

To examine the effect of nudge interventions in beverage kiosks on consumer drink purchases, participants were asked, “Did you notice any differences between the beverage kiosk you used and a typical kiosk?” and “Did the nudge interventions in the kiosk influence your beverage choice?” Responses to these items were recorded on a 7-point Likert scale (1 = strongly disagree, 7 = strongly agree) [45]. Additionally, using the same 7-point scale, participants rated the extent to which the nudge intervention facilitated healthier choices, as well as their attitudes toward, perceived necessity of, preferences for, and the extent to which they desired the implementation of such nudges in real-world beverage kiosks [45]. This study also examined which nudge interventions consumers perceived as most necessary for beverage kiosks, including the following: positioning non-SSBs at the front of the selection, expanding the variety of non-SSBs, combining both strategies (expanding the variety of non-SSBs and placing them at the front), or opting for no nudge intervention [43].

#### 2.5.4. Perception, Attitude, and Intention Toward Non-SSBs

To assess participants’ perceived importance of healthy eating, frequency of sweet food consumption, frequency of checking sugar content when purchasing processed foods, liking for non-SSBs, awareness of non-SSBs, attitudes toward non-SSBs, and purchase intentions, each construct was measured using a 7-point Likert scale (1 = strongly disagree, 7 = strongly agree) [45,46].

### 2.6. Data Analysis

All data were analyzed using IBM SPSS Statistics for Windows, Version 29.0. Frequency analysis was conducted to examine the demographic characteristics of the participants. One-way analysis of variance (ANOVA) was applied to identify the between-group differences in pre-existing conditions, kiosk usage experience, the awareness of kiosk differences, the influence of kiosk nudge interventions on beverage choices, perceived necessity, preferences, and willingness to implement nudges. Duncan’s Multiple Range Test was utilized for post hoc analysis at the 0.05 significance level. Independent samples t tests were performed to compare the differences based on sex in the awareness of kiosk differences, the influence of kiosk nudge interventions on beverage choice, the degree to which nudges supported healthier choices, attitudes toward nudges, perceived necessity, preferences, and the extent to which they desired their application in real-world settings. Chi-square tests (χ^2^) were employed to examine demographic characteristics, beverage selection, and the nudges that were perceived to be the most necessary for beverage kiosks based on sex and group. When more than 20% of the cells had expected frequencies less than 5 for the analysis of the demographic characteristics based on group, Fisher’s exact test was applied as an alternative. Binomial logistic regression was also conducted to assess the impact of kiosk nudge interventions on the purchase of non-SSBs, adjusting for sex and pre-existing conditions. To examine the effects of healthy eating behaviors and perceptions of non-SSBs on purchase intention, a multiple regression analysis was conducted using the stepwise method.

## 3. Results

### 3.1. Demographic Characteristics of the Participants

The demographic characteristics of the participants are summarized in Table 1. Females accounted for 81.3% of the participants, with single representing 99.5%. Regarding household type, 48.9% of participants were from three-person or larger households, while 45.6% were from single-person households.

### 3.2. Awareness and Impact of Beverage Kiosk Nudges

No significant differences were found in the demographic characteristics, pre-existing conditions, or beverage kiosk usage behaviors across the groups. Therefore, it was confirmed that participants were evenly and randomly assigned to the groups.

The group with the combination (order and variety) kiosk showed significantly greater awareness of the nudge intervention compared to other groups (*p* < 0.01). No significant differences in perception were found based on sex. Although there were no significant differences between groups in terms of the extent to which the nudge interventions influenced beverage selection, women were significantly more influenced than men (*p* < 0.05) (see Table 2).

### 3.3. Perceptions of Beverage Kiosk Nudges

The results detailing participants’ perceptions, attitudes, and perceived necessity regarding the nudges are presented in Table 3. Overall, women were more likely than men to believe that all three types of nudges would help facilitate healthier choices (*p* < 0.01). Additionally, women exhibited more positive attitudes toward all three types of nudges compared to men (*p* < 0.01). Women also reported a significantly higher preference for and desire to implement all three types of nudges; in addition, except for the single (order) nudge, they reported significantly higher perceptions of the necessity of the nudges compared to men. These differences ranged from small to large in magnitude (see Table 3 for Cohen’s d values), with the strongest effects observed for the variety nudge. By contrast, men did not show significant differences in perceived necessity, preference, or desire to implement regarding the three nudges. When asked which nudge was the most necessary for real-world beverage kiosks, participants selected the following: single (variety) (39.6%), combination (order and variety) (38.5%), and single (order) (20.9%). Among men, single (variety) was most frequently selected (38.2%), whereas women most often selected combination (order and variety) (41.2%) (*p* < 0.01).

### 3.4. Comparison of Beverage Kiosk Purchases

**Figure 3:**
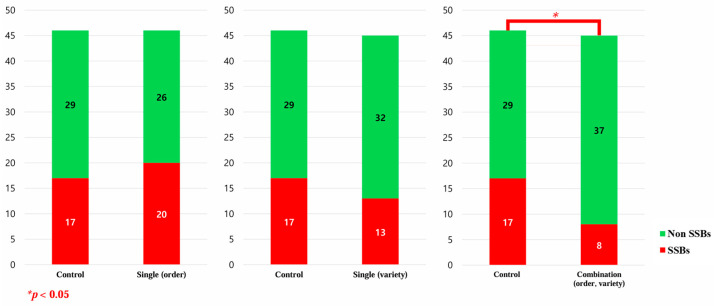
Comparison of beverage kiosk purchases between control group and beverage kiosk nudge groups. SSBs: sugar-sweetened beverages; non-SSBs: non-sugar-sweetened beverages.

Among the 14 beverage types sold in the kiosks in this study, 6 were classified as SSBs (Cola, lemon–lime soda, orange soda, chocolate drink, orange juice, lemonade), and 8 as non-SSBs (Diet cola, Diet lemon–lime soda, Diet orange soda, Diet chocolate drink, milk, 100% freshly squeezed orange juice, Diet lemonade, bottled water). The results comparing beverage purchases between the groups using the kiosks with nudge interventions and the control group are presented in Table 4 and Figure 3. The groups with the single (order) nudge kiosk (56.5%) and the single (variety) nudge kiosk (71.1%) did not lead to significantly higher non-SSB purchases compared to the control kiosk (63.0%). However, the purchase rate of non-SSBs for the group with the combination (order and variety) nudge kiosk (82.2%) was significantly higher than that for the control group (*p* < 0.05).

**Figure 4:**
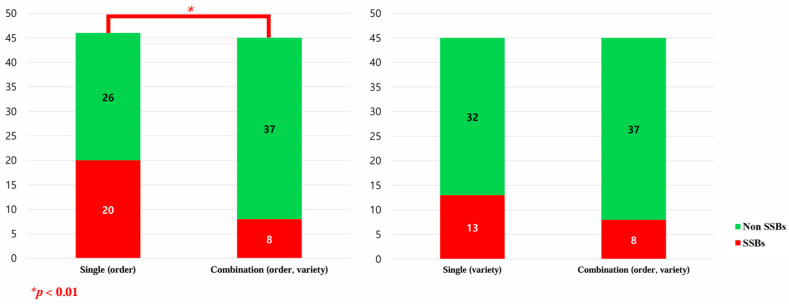
Comparison of beverage kiosk purchases between beverage kiosk nudge groups. SSBs: sugar-sweetened beverages; non-SSBs: non-sugar-sweetened beverages.

To investigate the effect of the combined nudge compared to that of the single nudge on non-SSB purchases, the results comparing beverage purchases between the groups using the kiosks with nudge interventions are presented in Table 5 and Figure 4. The purchase rate of non-SSBs in the group with the combination (order and variety) kiosk (82.2%) was significantly higher than that in the group with the single (order) kiosk (56.5%) (*p* < 0.01). However, there was no significant difference compared to the group with the single (variety) kiosk (71.1%).

### 3.5. Factors Influencing Non-Sugar-Sweetened Beverages

To examine the effect of nudge interventions on non-SSB purchases, the binomial logistic regression results are presented in Table 6, adjusting for participants’ sex and prior conditions. When sex and thirst level were the same, the group with the combination (order and variety) kiosk had 2.780-fold higher odds of purchasing non-SSBs compared to the control group (odds ratio [OR] = 2.780; 95% confidence interval [CI] = 1.035–7.468; *p* < 0.05). Therefore, the combination (order and variety) nudge can be considered effective in promoting non-SSB purchases regardless of sex or thirst level. Additionally, when the type of nudge and thirst level were controlled, females were 2.274 times more likely to purchase non-SSBs than males (odds ratio [OR] = 2.274; 95% confidence interval [CI] = 1.039–4.976; *p* < 0.05).

### 3.6. Influence of Dietary Behavior and Perception of Non-Sugar-Sweetened Beverages on Purchase Intention

The effects of perceived importance of healthy eating, frequency of sweet food consumption, frequency of checking sugar content on processed foods, liking for non-SSBs, awareness of non-SSBs, and attitudes toward non-SSBs on purchase intention is presented in Table 7. The regression model explained 55.3% of the variance in purchase intention and was statistically significant (*p* < 0.001). A stronger liking for non-SSBs (*p* < 0.001), more favorable attitudes toward non-SSBs (*p* < 0.001), and a higher frequency of checking sugar content in processed foods (*p* < 0.01) were significantly associated with greater intention to purchase non-SSBs instead of conventional sugar-sweetened beverages.

## 4. Discussion

Given the rise in global sugar intake from sugar-sweetened beverages (SSBs), promoting healthier beverage consumption is critical [47]. Freshly squeezed juices and artificially sweetened beverages (ASBs), which are free from added sugars, offer healthier alternatives [48]. This study investigates the effectiveness of single and combined nudges in promoting non-SSB purchases using kiosk setups designed to mimic typical beverage assortments and placement patterns in fast-food outlets.

Bar-Hillel’s research suggests that in simultaneous choice contexts like menu boards, items placed at the beginning or end are more likely to be selected [49]. Additionally, Schmidtke et al. found that fast-food consumers often choose the first-listed item on kiosks, assuming it is the most popular [30]. Likewise, expanding the variety of healthier options in sales environments can increase their perceived demand and attractiveness to consumers [50]. Moreover, it has also been shown that combining two or more nudges can further enhance consumer purchasing behavior. For instance, a study by Vellinga et al. demonstrated that combining price increases with an informational nudge about the environmental impact of meat production reduced meat purchases by 386 g compared to a control group [51]. Similarly, in their analysis of 26 experiments utilizing priming and salience nudges, Wilson et al. demonstrated the pronounced effectiveness of combined nudges over single nudges [52]. Thorndike et al. further found that using traffic light labels for cafeteria products, combined with increasing the visibility of healthier items and reducing the visibility of harmful ones, significantly boosted the sales of healthy items while reducing unhealthy purchases [53]. These findings collectively highlight the enhanced effectiveness of combined nudges in encouraging healthier food choices.

In this study, participants perceived the nudges as helpful in promoting healthier choices, with women rating them significantly higher in terms of both helpfulness and attitudes compared to men (*p* < 0.01). In particular, the single (variety) and combination (order and variety) nudges scored the highest in terms of perceived necessity, preference, and desire for real-world implementation. This finding suggests that consumers in their twenties prefer and perceive a greater need for a variety expansion nudge over an order nudge. Notably, women rated all aspects of the nudges significantly higher—except for the perceived necessity of the single (order) nudge. Overall, women also exhibited more positive perceptions of and attitudes toward kiosk nudges in general and expressed greater demand for their implementation. These results align with research by Krisam et al. which indicated that German women reported significantly higher acceptance levels for seven types of nudges compared to men [54], and Reisch et al. which, similarly, identified greater nudge acceptance among women [55]. Women were more strongly influenced by nudges (*p* < 0.05) and reported stronger perceptions of their necessity and more positive attitudes toward them overall in this study.

The purchase data from actual beverage kiosks revealed no significant effects for either of the single nudges (order or variety) when compared to the control group. However, the combination (order and variety) nudge significantly increased non-SSB purchases compared to the control group (*p* < 0.05) and was more effective than the single (order) nudge (*p* < 0.01). Moreover, under identical conditions of sex and thirst level, the odds of purchasing non-SSBs were 2.780 times higher in the combination (order and variety) nudge condition compared to the control (*p* < 0.05). This finding suggests that expanding the variety of non-SSBs and placing them at the front are indeed effective in promoting their purchase. However, the relatively wide confidence interval (CI = 1.035–7.468) indicates a degree of imprecision in the estimated odds ratio. While the direction of the effect appears consistent, the magnitude should be interpreted with caution. Future studies employing larger sample sizes or repeated exposure designs are warranted to enhance the precision and robustness of effect size estimates.

The limited scope of kiosk screens compared to physical store shelves or menu boards may make it challenging for consumers to notice changes in beverage placement order. However, expanding the variety of healthier beverages can increase their visibility on the kiosk screen, which may lead consumers to perceive them as more prevalent and popular. Research by Stamos et al. suggests that emphasizing healthiness through nudges such as expanding healthier product options helps consumers justify healthier choices, thereby facilitating actual purchases [56]. Additionally, when the visibility of healthier options increases on the kiosk screen, rearranging their order may become more noticeable, leading to the highest purchase rates when combined with variety expansion. Indeed, the synergy of combined nudges in maximizing healthy choice promotion has been supported by prior studies. For instance, Hoenink et al. reported increased healthy food purchases when price-change and informational nudges were combined in a virtual supermarket [57]. Similarly, in a university dining setting, Vermote et al. observed that fruit purchases significantly increased over four weeks as nudges were cumulatively added, with sustained effects over time [58]. Therefore, implementing strategies that combine two or more nudges, with a focus on variety expansion are particularly successful in encouraging healthy choices among consumers.

In this study, under identical kiosk and thirst level, women had 2.274 times higher odds of purchasing non-SSBs than men (*p* < 0.05). This is consistent with previous findings that women are more likely than men to practice healthy eating [59] and more sensitive to the seriousness of harmful food consumption [60]. Additionally, women are known to have a greater need and purchase intention for healthy foods compared to men [61]. These gender-based differences may not be purely behavioral but may instead reflect deeper psychological and sociocultural mechanisms. Prior studies have shown that women tend to be more sensitive to health-related risks [62], possess higher self-efficacy in healthy eating [63], and are more likely to critically monitor and evaluate their health-related behaviors in accordance with internalized social norms [64]. Considering these findings, women not only report a higher need and intention for healthy food consumption but also demonstrate significantly higher actual purchase rates for healthy foods. Given women’s higher responsiveness to certain nudging strategies in this study, incorporating their preferences may enhance intervention effectiveness in relevant contexts. However, to avoid overgeneralization and ensure broader applicability, future strategies should also explore nuanced approaches that consider diversity within and across gender groups, including sociocultural and behavioral factors that influence beverage choices. In line with this, combination nudges may be particularly effective when applied in environments frequently visited by women, such as cafés [65], where their preferences and behavioral tendencies can be more directly reflected.

Meanwhile, Korean men prioritize convenience and speed in their food choices, frequently purchasing meals at convenience stores [66]. Korean male college students, in particular, tend to buy beverages at convenience stores [38], often pairing their meal purchases with carbonated drinks [61]. To support non-SSB consumption among men, it may be helpful to develop carbonated alternatives that provide similar sensory satisfaction to traditional sugary beverages. Moreover, as men in this demographic have reported greater sensitivity to taste and price in prior study [67], strategies such as price promotions or in-store incentives could be effective. Some studies have found that men tend to show lower perceived need for sugar-reduced products and report less intention to purchase or recommend them [9,68]. To address this, providing visual labeling that highlights sugar content or incorporating educational prompts about the health impact of added sugars within purchase environments may help increase awareness and support informed decision-making. While these gender-based patterns offer valuable insights for tailoring interventions, it is important to interpret them with caution to avoid reinforcing gender-stereotyped behavioral expectations. Future research and public health strategies should adopt a more inclusive and gender-sensitive framework, considering the intersection of gender with other sociodemographic factors such as age, cultural norms, and health literacy. This approach can help ensure that nudging interventions are equitably designed and effective across diverse population groups.

The present study examined the influence of health eating behaviors and perceptions of non-SSBs on purchase intention. The results indicated that more favorable attitudes toward non-SSBs (*p* < 0.001), stronger liking for non-SSBs (*p* < 0.001), and more frequent checking of sugar content in processed foods (*p* < 0.01) were significantly associated with increased purchase intention. Prior research has shown that favorable attitudes toward food products are positively associated with increased purchase intention [61]. Therefore, to increase consumer adoption of non-SSBs, companies should implement strategic marketing efforts to foster positive perceptions and attitudes toward these products. It is also necessary to identify the sources of negative perceptions and dissatisfaction regarding non-SSBs and develop appropriate solutions. In particular, key product attributes such as taste and price—factors that consumers consider important in purchasing decisions—should be carefully examined and enhanced [69,70]. Moreover, implementing visually salient sugar labeling or placing visual cues that highlight the importance of sugar reduction in purchasing environments may help induce behavioral change by encouraging consumers to check sugar content more intuitively.

The findings of this study provide policy-relevant insights that can guide the development of concrete strategies to promote non-SSBs consumption in digital food environments. According to the results, increasing the proportion of non-SSBs and positioning them at the top of kiosk menus may enhance their visibility and likelihood of selection. These findings may inform the formulation of evidence-based interface design standards for digital ordering systems. In this study, approximately 57% of the available beverage options were classified as non-SSBs; this proportion could serve as an initial benchmark for healthier menu compositions in future policy frameworks. To determine optimal configurations, follow-up studies and pilot applications are needed across diverse consumer settings and target populations. For instance, implementing combination nudges in high-traffic environments such as university food courts may support context-sensitive evaluations of policy feasibility. Grounded in the behavioral and perceptual outcomes observed in this study, these recommendations offer a practical basis for integrating nudge-informed interface policies into broader efforts to foster healthier food and beverage environments through digital platforms.

### 4.1. Strength

This study offers applied evidence on how nudging strategies can promote non-sugar-sweetened beverage (non-SSB) consumption in real-world settings. To ensure ecological validity, the experiment was conducted in a fully replicated kiosk environment where participants physically interacted with the interface and completed beverage purchases using their own credit cards. We compared the effects of order and variety nudges on non-SSB purchases, providing meaningful data for beverage consumption settings. Beyond behavioral outcomes, this study also assessed user-centered indicators such as perceived effectiveness, necessity, preference, and willingness to implement each nudging strategy. This multidimensional approach allows for a more comprehensive understanding of both the actual and perceived impacts of nudges. Furthermore, regression analysis of psychological variables related to non-SSB purchase intention offers practical insights for future intervention design. By combining behavioral, perceptual, and attitudinal data in a realistic consumption context, this study provides an applied foundation for developing user-aligned, context-sensitive nudging strategies in food service environments.

### 4.2. Limitation

Despite its strengths and practical implications, this study has several limitations that should be acknowledged. First, there are issues related to participant characteristics. A substantial majority of participants were female (81.3%), which reflects the demographic profile of the study location—an area near a women’s university—and resulted in a limited representation of male consumers. This imbalance may have influenced the observed receptivity to the nudging interventions, as female participants demonstrated greater responsiveness and a higher tendency to select non-SSBs. To statistically address this imbalance, sex was included as a covariate in the logistic regression analysis. The combination nudge remained a significant predictor even after this adjustment, suggesting that the intervention effect was not solely attributable to sample composition. However, statistical adjustment cannot fully substitute for balanced sampling. To improve gender representativeness and ensure that behavioral responses across sexes are meaningfully compared, future research should aim to recruit a more balanced number of male and female participants. In addition, while participants’ income levels were collected for demographic profiling, subgroup analyses by socioeconomic status were not conducted, as this study focused primarily on behavioral responses to nudge interventions and gender-based differences. Given the potential for differential responses across income groups, future research should explore the moderating role of socioeconomic status to ensure effective implementation of nudging strategies. Furthermore, as this study was conducted only with Korean adults aged 19–29 living in an urban setting, the findings may not generalize to other age, cultural, or regional groups. To enhance external validity, future research should include a broader range of demographic populations. Second, even though participants were unaware of this study’s true purpose and kiosk settings until after completing their purchases, those in the combination nudge group—which exhibited the most pronounced behavioral effect—were significantly more likely to recognize the manipulation. This raises the possibility that the observed effect was partially driven by participant awareness, introducing demand characteristics. Moreover, despite efforts to replicate typical commercial kiosk environments, participants may have noticed cues suggesting a research context—such as the absence of brand signage or the presence of informed consent procedures. These factors may have heightened their awareness that they were part of a study rather than making routine purchases, potentially influencing their responses. Additionally, although the kiosk interface closely resembled real-world ordering systems, certain contextual elements—such as promotional signage or the presence of other customers—were not incorporated. These omissions may have limited the ecological validity of this study and reduced the generalizability of the findings to actual consumer behavior in commercial settings. Given these potential biases, future research should implement observational designs in real-world consumer settings—such as supermarkets or fast-food restaurants—to better evaluate the effectiveness and ecological validity of nudging interventions. Third, this study assessed participants’ beverage choices based on a single purchase occasion, which constrains the ability to draw conclusions about sustained behavioral change or long-term health outcomes. Future research incorporating repeated exposures or longitudinal follow-up is recommended to more accurately evaluate the durability and real-world effectiveness of nudge interventions. Fourth, although the kiosk interface was consistent across all conditions (e.g., color, button size, and overall screen design), we did not conduct a formal visual audit or assess user experience factors. As such, participants’ choices may have been partially influenced by visual prominence. Future research incorporating eye-tracking or user feedback could help disentangle these effects. Lastly, while this study provided empirical evidence supporting the effectiveness of a combined nudge strategy in digital food environments, it lacked a fully elaborated theoretical account of how such combinations influence behavior. Although the design prioritized ecological validity and practical applicability, the absence of a clearly defined cognitive or behavioral mechanism may limit the theoretical generalizability of the findings. Future research should incorporate theory-driven models to elucidate the mechanisms by which combined nudges operate in digital food purchasing contexts. Nonetheless, a key strength of this study lies in its ecologically grounded design. By replicating real-world kiosk interfaces and measuring actual consumer purchases, this study extends beyond hypothetical scenarios and contributes practical insights with strong external validity.

## 5. Conclusions

Nudge interventions through real-world kiosk screens can be an effective approach to mitigating excessive sugar intake, particularly among young adults in their 20 s. Strategies that expand the variety of healthier beverages and position them more prominently have been shown to significantly enhance actual purchases. Notably, women demonstrated greater receptiveness and a more favorable attitude toward these nudge strategies compared to men, and they were also more likely to opt for healthier choices. These findings highlight the necessity of developing gender-specific strategies to promote and market non-SSBs. Future public health efforts should prioritize reducing sugar intake, and further research is warranted to evaluate the real-world effectiveness of nudge strategies.

## Figures and Tables

**Table 1:** Demographic profile of subjects.

Variable	*N* (%)
Sex	
Male	34 (18.7)
Female	148 (81.3)
Marriage status	
Married	1 (0.05)
Single	181 (99.5)
Household type	
Single	83 (45.6)
Two-person	10 (5.5)
Three-person & over	89 (48.9)
Monthly income/Allowance (in KRW 10,000)	
<20	5 (2.7)
20–40	14 (7.7)
40–60	48 (26.4)
60–80	44 (24.2)
80–100	23 (12.6)
100–200	28 (15.4)
>200	20 (11.0)
Current weight management status	
Trying to lose weight	89 (48.9)
Trying to maintain weight	43 (23.6)
Trying to gain weight	10 (5.5)
Not trying to gain or lose weight	40 (22.0)

**Table 2:** Awareness and impact of beverage kiosk nudges.

	Total (*n* = 182)	Single (Order) (*n* = 46)	Single (Variety) (*n* = 45)	Combination (Order, Variety) (*n* = 45)	F/*P*	Male (*n* = 34)	Female (*n* = 148)	t/*p*
	Mean ± SD	Mean ± SD	Mean ± SD	Mean ± SD		Mean ± SD	Mean ± SD	
Awareness of kiosk differences	3.14 ± 1.93	2.98 ± 1.78 ^a^	2.56 ± 1.83 ^a^	3.89 ± 1.97 ^b^	6.038 **	2.96 ± 2.09	3.18 ± 1.90	−0.514
Impact of kiosk features on beverage choice	3.62 ± 1.83	3.30 ± 1.76	3.76 ± 1.86	3.80 ± 1.85	1.030	2.76 ± 1.94	3.81 ± 1.75	−2.487 *

* *p* < 0.05, ** *p* < 0.01, post hoc analysis using Duncan’s test: ^a^ < ^b^.

**Table 3:** Attitudes, preferences, desire to implement, and perceived necessities for beverage kiosk nudges.

	Total (*n* = 182)	Male (*n* = 34)	Female (*n* = 148)	t/*p*	Cohen’s d
	Mean ± SD	Mean ± SD	Mean ± SD		
Effectiveness of nudge in promoting healthier choice	5.05 ± 1.58	4.27 ± 2.02	5.23 ± 1.41	−3.303 **	−0.623
Attitude toward nudges	5.84 ± 1.16	5.29 ± 1.29	5.96 ± 1.09	−3.089 **	−0.593
Necessity of nudges					
Single (order)	5.53 ± 1.26 ^a^	5.27 ± 1.19	5.59 ± 1.27 ^a^	−1.356	−0.255
Single (variety)	6.09 ± 1.05 ^b^	5.44 ± 1.50	6.24 ± 0.85 ^b^	−4.198 ***	−0.799
Combination (order, variety)	5.91 ± 1.08 ^b^	5.50 ± 1.33	6.00 ± 1.00 ^b^	−2.494 *	−0.468
F	11.835 ***	0.281	14.671 ***		
Preference for nudges					
Single (order)	5.62 ± 1.29 ^a^	5.18 ± 1.45	5.72 ± 1.23 ^a^	−2.254 *	−0.424
Single (variety)	6.10 ± 1.15 ^b^	5.44 ± 1.54	6.26 ± 0.98 ^b^	−3.874 ***	−0.743
Combination (order, variety)	5.96 ± 1.10 ^b^	5.44 ± 1.28	6.08 ± 1.02 ^b^	−3.135 **	−0.597
F	8.039 ***	0.390	9.308 ***		
Desire for nudge implementation					
Single (order)	5.66 ± 1.29 ^a^	5.24 ± 1.71	5.76 ± 1.15 ^a^	−2.155 *	−0.409
Single (variety)	6.14 ± 1.14 ^b^	5.53 ± 1.59	6.28 ± 0.96 ^b^	−3.600 ***	−0.680
Combination (order, variety)	5.94 ± 1.17 ^b^	5.44 ± 1.56	6.05 ± 1.04 ^b^	−2.803 **	−0.529
F	7.453 **	0.296	9.321 ***		
	***N* (%)**	***N* (%)**	***N* (%)**	**χ^2^/*p***	
Most necessary nudge in beverage kiosk				11.745 **	
Single (order)	38 (20.9)	10 (29.4)	28 (18.9)		
Single (variety)	72 (39.6)	13 (38.2)	59 (39.9)		
Combination (order, variety)	70 (38.5)	9 (26.5)	61 (41.2)		

* *p* < 0.05, ** *p* < 0.01, *** *p* < 0.001, post hoc analysis using Duncan’s test: ^a^ < ^b^. Effect sizes are reported as Cohen’s d with male as the reference group; negative values reflect higher female scores.

**Table 4:** Comparison of beverage kiosk purchases between control group and beverage kiosk nudge groups.

	SSBs	Non-SSBs	χ^2^/*p*
	*N* (%)	*N* (%)	
Control (n = 46)	17 (37.0)	29 (63.0)	0.407
Single (order) (n = 46)	20 (43.5)	26 (56.5)	
Control (n = 46)	17 (37.0)	29 (63.0)	0.670
Single (variety) (n = 45)	13 (28.9)	32 (71.1)	
Control (n = 46)	17 (37.0)	29 (63.0)	4.199 *
Combination (order, variety) (n = 45)	8 (17.8)	37 (82.2)	

* *p* < 0.05. SSBs: sugar-sweetened beverages; non-SSBs: non-sugar-sweetened beverages.

**Table 5:** Comparison of beverage kiosk purchases between beverage kiosk nudge groups.

	SSBs	Non-SSBs	χ^2^/*p*
	*N* (%)	*N* (%)	
Single (order) (n = 46)	20 (43.5)	26 (56.5)	7.053 **
Combination (order, variety) (n = 45)	8 (17.8)	37 (82.2)	
Single (variety) (n = 45)	13 (28.9)	32 (71.1)	1.553
Combination (order, variety) (n = 45)	8 (17.8)	37 (82.2)	

** *p* < 0.01 SSBs: sugar-sweetened beverages; non-SSBs: non-sugar-sweetened beverages.

**Table 6:** Logistic regression for dependent variable non-sugar-sweetened beverages.

Model	Independent Variable		B	SE	Wald	*p*	OR	95% CI	
								LLCI	ULCI
Model (Adjusted)	Nudges	Single (order)	−0.294	0.434	0.458	0.499	0.745	0.318	1.746
		Single (variety)	0.361	0.454	0.632	0.426	1.435	0.589	3.497
		Combination (order, variety)	1.022	0.504	4.113	0.043 *	2.780	1.035	7.468
	Sex	Female	0.822	0.399	4.230	0.040 *	2.274	1.039	4.976
	Thirst		0.053	0.145	0.134	0.714	0.688	0.794	1.401

Reference group: Nudges * Control kiosk, Sex * Male. * *p* < 0.05.

**Table 7:** Influence of dietary behavior and perception of non-sugar-sweetened beverages on purchase intention.

Dependent Variable	Independent Variable	B	SE	*β*	t(*p*)	TOL	VIF
Intention to purchase non-SSBs	(Constant)	1.334	0.352		3.787		
	Liking for non-SSBs	0.376	0.057	0.436	6.610 ***	0.567	1.764
	Attitude toward non-SSBs	0.399	0.075	0.334	5.293 ***	0.620	1.613
	Checking sugar content in processed foods	0.078	0.032	0.131	2.452 **	0.868	1.152
F(*p*) = 75.572 ***, R^2^ = 0.560, adj. R^2^ = 0.553							

** *p* < 0.01, *** *p* < 0.001. Non-SSBs: non-sugar-sweetened beverages.

## Data Availability

The datasets used and/or analyzed during the current study are available from the corresponding author on reasonable request.

## Abbreviations

The following abbreviations are used in this manuscript:

SSBs	Sugar-sweetened beverages
Non-SSBs	Non-sugar-sweetened beverages
